# Replacement of Sublineages of Avian Influenza (H5N1) by Reassortments, Sub-Saharan Africa

**DOI:** 10.3201/eid1411.080555

**Published:** 2008-11

**Authors:** Ademola A. Owoade, Nancy A. Gerloff, Mariette F. Ducatez, Jolaoso O. Taiwo, Jacques R. Kremer, Claude P. Muller

**Affiliations:** University of Ibadan, Ibadan, Nigeria (A.A. Owoade); National Public Health Laboratory, Luxembourg (N.A. Gerloff, M.F. Ducatez, J.R. Kremer, C.P. Muller); Ogun State Ministry of Agriculture, Abeokuta, Nigeria (J.O. Taiwo); 1 These authors contributed equally to this article.; 2 Current affiliation: St. Jude Children's Research Hospital, Memphis, Tennessee, USA.

**Keywords:** Highly pathogenic avian influenza virus, subtype H5N1, reassortment, Nigeria, NS gene, research

## Abstract

Eight new full-length sequences from highly pathogenic avian influenza viruses (H5N1) from 4 states in southwest Nigeria were analyzed. All gene sequences were more closely related to the first strains found in Nigeria in 2006 than to any strain found outside the country. Six viruses had evolved by at least 3 reassortment events (AC_HA/NS_, AC_NS_) from previously identified sublineages A (EMA 2) and C (EMA 1). Our results suggest that highly pathogenic avian influenza viruses (H5N1) initially imported into Nigeria in 2006 have been gradually replaced by various reassortments. In all reassortants, nonstructural genes were derived from sublineage C with 2 characteristic amino acids (compared with sublineage A). If the high prevalence of reassortants was typical for West Africa in 2007, the absence of such reassortants anywhere else suggests that reintroductions of influenza A (H5N1) from Africa into Eurasia must be rare.

## Abstract 2

The 3 sublineages initially introduced into Nigeria in 2006 were gradually replaced by different reassortants.

Highly pathogenic avian influenza (HPAI) virus subtype H5N1 in Africa was first reported from northern Nigeria in February 2006. Phylogenetic analysis of the complete genome showed that these viruses were clearly distinct from the 2 lineages that were found during the same period in southwestern Nigeria (*1*,*2*). The 3 sublineages (referred to as A, B, and C), 2 of which emerged from a common node, had evolved from subtype H5N1 strains that were originally found around Qinghai Lake in 2005. These strains clustered with viruses isolated from 2006 from southern Russia, Europe, and the Middle East (clade 2.2, www.who.int/csr/disease/influenza/tree_large.pdf) but not with the strains prevalent in southeast Asia (*3*). The timeline, the observed influenza A (H5N1) substitution rates in Africa, and the phylogenetic relationship suggested that the sublineages were independently introduced into the country (*1*,*2*). These sublineages were later found throughout Africa with a distinct geographic distribution (*2*,*4*). Sublineage A was also found in Niger and Togo (hemagglutinin [HA] sequence); sublineage B was detected in Egypt and in a human patient in Djibouti (partial HA sequence), and sublineage C was found in Burkina Faso, Sudan, Côte d’Ivoire, Ghana (HA and neuraminidase [NA] sequences) (*5*) and Cameroon (NA sequence) (*6*). Sublineage A strains were also referred to as EMA 2, and both sublineages B and C belong to EMA 1 (*3*). In 2006, one strain with reassorted genes was reported among 35 full-length sequences of the European–Middle Eastern–African lineage (*1*–*4*). We describe new HPAI (H5N1) strains collected in southwestern Nigeria during the second half of 2007, most of which were different reassortants of sublineages A and C.

## Materials and Methods

Cloacal swabs were obtained from 8 chicken farms in Lagos (*1*), Ogun (5), Oyo (*1*) and Ekiti (1) States from June through November 2007. RNA extraction from cloacal swabs, reverse transcription–PCR amplification, and gene sequencing were conducted as described (*1*). For most viruses, complete sequences were obtained for all gene segments. Kimura distances were calculated on the basis of complete or partial gene sequences by including the maximum sequence length available from all strains included in the comparison. Phylogenetic trees were calculated by using PAUP version 4.0 beta 10 (*7*) with the maximum-likelihood method. The best model was determined by using MODELTEST (*8*). The sequences have been submitted to GenBank with the accession nos. FM160635–FM160642 and FM164800–FM164855.

## Results

### Reassortants

**Figure 1:**
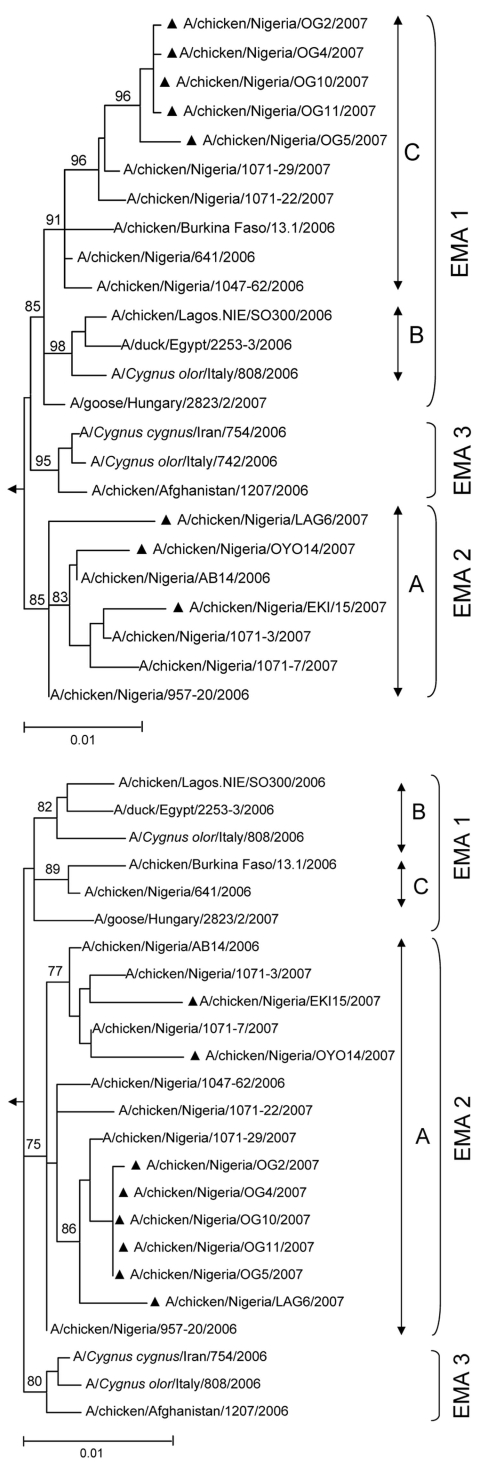
Phylogeny of hemagglutinin (A) and neuraminidase (B) genes from 8 HPAI (H5N1) viruses collected in Nigeria during the second half of 2007 (▲), in comparison with previously identified sublineage A (EMA 2), sublineage B and C (EMA 1), and (EMA 3) strains (*1**,**3*)*.* The tree was calculated by using the maximum likelihood method implemented in PAUP 4.0 (*7*). The substitution model was obtained by using MODELTEST (*8*). Bootstrap values (%) were calculated with the maximum-likelihood method with 1,000 replications and are indicated on key nodes. Scale bars represent ≈1% of nucleotide changes between close relatives. A/duck/Anyang/AVL-1/2001 was used as an outgroup.

**Figure 2:**
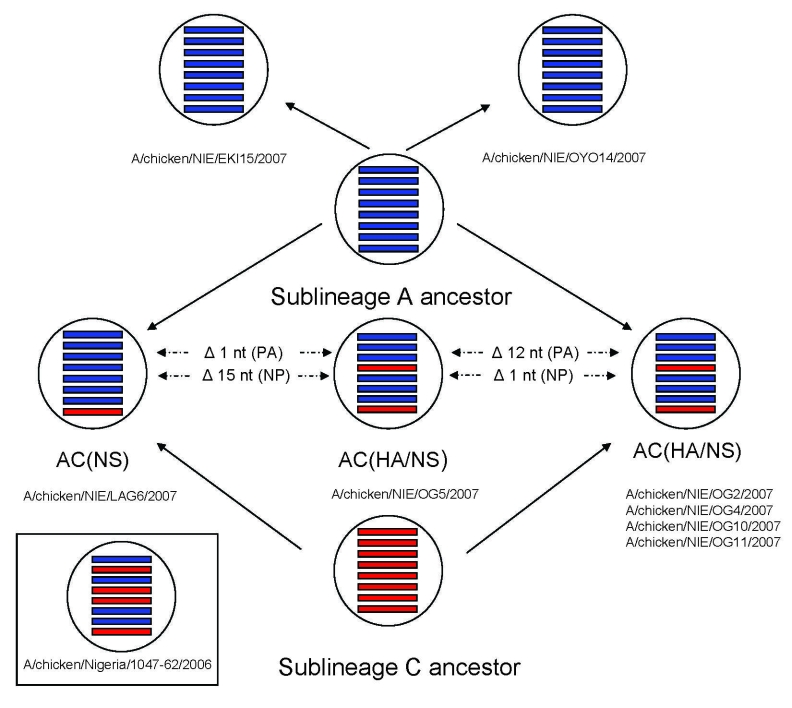
Schematic presentation of sublineage A–derived highly pathogenic avian influenza viruses (H5N1) and reassortants of sublineage A– and sublineage C–derived viruses identified in Nigeria in 2007. The reassortant reported from Salzberg and others in 2007 (3) is also shown. Sublineage A–derived gene segments are shown in blue; sublineage C–derived gene segments are shown in red. Gene segments are represented in the following order (from top): PB2, PB1, PA, HA, NP, NA, M, NS.

All genes of A/chicken/NIE/EKI15/2007 and A/chicken/NIE/OYO14/2007 clustered phylogenetically with sublineage A strains (Figures 1, 2). The Kimura distances between the genes of these viruses were 0.4%–1.4%. Among all subtype H5N1 virus sequences published in the Influenza Sequence Database (*5*), NIE/EKI15/2007 and NIE/OYO14/2007 gene sequences were most closely related to those found throughout 2006 and 2007 in Nigeria. Thus, these viruses have most probably evolved from a sublineage A virus initially imported into the country in 2006. This finding is also corroborated by published substitution rates from Africa (*2*).

Five viruses had HA and nonstructural (NS) genes grouping with sublineage C virus genes, whereas the other gene segments were most closely related to sublineage A viruses (e.g., A/chicken/NIE/OG2/2007 and OG5/2007, Figures 1, 2). These viruses evolved by reassortment from sublineages A and C viruses (AC_HA/NS_ reassortment, Figure 2).

Another virus (A/chicken/NIE/LAG6/2007) also showed evidence of reassortment between sublineage A and sublineage C. However, in this virus only the NS gene belonged to sublineage C (Figures 1, 2). The other 7 gene segments of A/chicken/NIE/LAG6/2007 were derived from sublineage A (AC_NS_ reassortant).

### Reassortments between Reassortants

Four of the AC_HA/NS_ reassortants (A/chicken/NIE/OG2/2007, A/chicken/NIE/OG4/2007, A/chicken/NIE/OG10/2007, and A/chicken/NIE/OG11/2007), all of which were from Ogun State, had similar sequences in all genes (Kimura distances 0%–0.7 %). The AC_NS_ reassortant A/chicken/NIE/LAG6/2007, obtained from a chicken farm in Lagos State, diverged by 0.9 % in the complete NS gene (derived from C lineage) and by 0.7% to 1.4 % in sublineage A–related gene segments from the latter 4 AC_HA/NS_ reassortants. Some gene segments of the AC_HA/NS_ reassortant A/chicken/NIE/OG5/2007 were most closely related to the other 4 AC_HA/NS_ reassortants, whereas, other gene segments were closer to the AC_NS_ reassortant A/chicken/NIE/LAG6/2007. Matrix protein, HA, NS, NA, and nucleocapsid protein (NP) genes of NIE/OG5/2007 showed a maximal Kimura distance of only <0.4% to AC_HA/NS_ reassortant genes but a distance of 0.6%–1.5% to the AC_NS_ reassortant (A/chicken/NIE/LAG6/2007). In contrast, RNA polymerase B protein (PB2), PB1, and PA genes were more closely related to the AC_NS_ reassortant (maximum Kimura distance: <0.6%) than to AC_HA/NS_ reassortants (minimum Kimura distance for the different genes: 0.7%–0.8%). For instance, A/chicken/NIE/OG5/2007 differed by 12 nucleotides in the PA gene from the most closely related AC_HA/NS_ reassortant (A/chicken/NIE/OG2/2007) but by only 1 nucleotide from the AC_NS_ reassortant (A/chicken/NIE/LAG6/2007). On the other hand, A/chicken/NIE/OG5/2007 had 15 nucleotides in the NP gene different from the A/chicken/NIE/LAG6/2007 but only 1 nucleotide difference compared with the closest AC_HA/NS_ reassortant (A/chicken/NIE/OG11/2007) (Figure 2). This finding strongly suggests that A/chicken/NIE/OG5/2007 is the result of an additional reassortment event involving an exchange of genes between the AC_HA/NS_ and AC_NS_ reassorted viruses.

### Mutations

The amino acid sequences of the HA cleavage site (PQGERRRKKRG) of the strains described here are identical to those of all HPAI (H5N1) strains reported from West Africa. All viruses had identical amino acids in all positions of the HA protein that are associated with preferential binding to α2,3-linked sialic acid (*9*,*10*) as described (*2*).

As for all HPAI (H5N1) strains from Africa, the above viruses had the virulence marker lysine (K) in position 627 of PB2 associated with accelerated viral replication, reduced host defense, higher mortality rate in mice (*11*), and a wider host range of subtype H5N1 strains (*12*). None of the known markers in the matrix 2 gene associated with resistance to amantadine (*13*) and in the NA gene associated with resistance to oseltamivir (H274Y) (*14*) were detected.

## Discussion

Gene sequences of all 8 HPAI viruses (H5N1) described here were more closely related to sublineages A or C strains found in Nigeria than to any other published H5N1 virus subtypes. In particular, they were more closely related to the first strains found in Nigeria in the beginning of 2006 than to any strains found outside the country. Thus, the viruses detected in southwestern Nigeria during the second half of 2007 probably evolved from the first viruses brought into the country in early 2006 (*1*), suggesting that HPAI (H5N1) has continuously circulated and is endemic to Nigeria. Sublineage A viruses have continued to circulate in Nigeria, whereas sublineage B was found only once on 1 farm (SO layer farm, Lagos, January 2006), and sublineage C viruses were no longer detected in 2007. Sublineage A viruses have been detected in northeastern Nigeria in February 2007 (*15*) and in 2 states of southwestern Nigeria during the last quarter of 2007 (A/chicken/NIE/EKI15/2007 and A/chicken/NIE/OYO14/2007). Sublineages B and C viruses may have been eliminated in Nigeria by effective countermeasures.

All AC_HA/NS_ described here were obtained from chicken flocks in Ogun State from June through August 2007. These results are similar to those found in the beginning of 2007 in other states of Nigeria (*15*). In addition, we identified an AC_NS_ reassortant in Lagos State (A/chicken/NIE/LAG6/2007) distinct from the latter strain. At least 2 distinct reassortment events were necessary to generate sublineages A and C reassortants AC_HA/NS_ and AC_NS,_ which probably had occurred already in 2006, as suggested by the conspicuous absence of sublineage C in 2007. Although it is obviously more difficult to demonstrate reassortment events between genetically similar viruses, the asymmetry in gene divergence of A/chicken/NIE/OG5/2007 compared with the other AC_HA/NS_ and AC_NS_ reassortants suggests that additional reassortment events have taken place.

In 2006, only 1 reassorted strain was found among 35 European–Middle Eastern– African strains, including 19 viruses reported from Nigeria, belonging to 3 parent sublineages (*1*–*4*). In the beginning of 2007, 10 of 12 from northern, southern, and central states all belonged to the same AC_HA/NS_ reassortants (*15*), distinct from the AC_PB1/HA/NP/NS_ reassortant detected in 2006 (*3*). Similar reassortants were also found in other regions of sub-Saharan Africa (unpub. data). During the second half of 2007, we found 6 reassortants including 3 distinct reassortants among 8 strains collected from 8 farms located in 4 contiguous Federal States of Nigeria (Figure 2). These results suggest that reassortants have largely replaced the initial sublineages from which they were derived and that reassortments are pervasive. This finding confirms that reassortments between subtype H5N1 viruses occur frequently when different strains cocirculate in the same region (*16*) and is of particular concern if the increasing prevalence is the result of adaptation to the African environment.

Although segments of the replication complex (PB1, PB2, PA, and NP) may reassort individually without affecting viral fitness (*16*), there seems to be a coordinated evolution of the HA and NA genes (*17*). In all but 1 of the Nigerian reassortants, HA and NA genes originated from different sublineages (C and A), suggesting compatibility between phenotypes of both sublineages. All reassortants from Nigeria included sublineage C–derived NS genes, which may suggest a higher fitness of these viruses. Sublineage C–derived NS1 and NS2 sequences from all Nigerian reassortants and 11 unpublished sequences from AC_HA/NS_ reassortants identified in other sub-Saharan regions showed 2 amino acids (NS1 V194 and NS2 R34), which were never identified in sublineage A viruses. It has been shown that modifications in the NS proteins, including amino acids adjacent to V194, may modulate the virulence of HPAI (H5N1) (*18*,*19*). Alternatively, the observation that all reassortants in West Africa have sublineage C–derived NS genes may suggest a better adaptation to the African environment of viruses that came from the cold temperatures of central Asia. Thus, the influence of differences in ecology between Africa and Eurasia on viral selection and dynamics deserves further attention.

Although no reassortments have been reported among clade 2.2 viruses (www.who.int/csr/disease/influenza/tree_large.pdf) in Central Asia, Europe, and the Middle East since their emergence from Qinghai Lake region in 2005, reassortments of these viruses seem to be rampant in sub-Saharan Africa, where they have become the critical determinant of genetic diversity of HPAI (H5N1). Because of low prevalence, mainly in wild birds, clade 2.2 viruses have few opportunities to reassort in Eurasia. In contrast, opportunities to reassort seem to be frequent in sub-Saharan Africa because of great difficulties in setting up a sensitive surveillance system in a complex socioeconomic environment, where backyard farms and large commercial farms with variable biosafety levels coexist, and where culling may threaten the livelihood and survival of the farm.

If the high prevalence of reassortants was typical for West Africa in 2007, the absence of such reassortants anywhere else suggests that reintroductions of subtype H5N1 from Western Africa into Eurasia must be rare. Moreover, all HPAI (H5N1) strains from Nigeria in 2007 were more similar to those found in Nigeria in 2006 than to even the closest relative from Europe in 2007 (Hungary). Although subtype H5N1 has been found in wild birds from Africa, such as vultures (*4*), HPAI (H5N1) has so far not been reported in long-distance migrating birds in West Africa. Thus, the exchange of subtype H5N1 between Eurasia and Africa seems to be a rare event, which in 2006 may have been triggered by unusual bird migration as a result of the central Asian cold spell.

The biological significance of reassortments between genetically similar viruses may be arguable, but the frequency of reassortment events is an important marker of virus endemicity in a region. Moreover, endemicity of HPAI (H5N1) and a high propensity of reassorting in a region where seasonal influenza is unchecked are essential ingredients of the anticipated pandemic.
